# Nanoscale integration of single cell biologics discovery processes using optofluidic manipulation and monitoring

**DOI:** 10.1002/bit.27024

**Published:** 2019-06-24

**Authors:** Marsela Jorgolli, Tanner Nevill, Aaron Winters, Irwin Chen, Su Chong, Fen‐Fen Lin, Marissa Mock, Ching Chen, Kim Le, Christopher Tan, Philip Jess, Han Xu, Agi Hamburger, Jennitte Stevens, Trent Munro, Ming Wu, Philip Tagari, Les P. Miranda

**Affiliations:** ^1^ Amgen Research One Amgen Center Drive Thousand Oaks California; ^2^ Product Applications Berkeley Lights, Inc Emeryville California; ^3^ Drug Substance Technologies, One Amgen Center Drive Thousand Oaks California; ^4^ Drug Discovery A2 Biotherapeutics Westlake Village California; ^5^ Department of Electrical Engineering and Computer Sciences University of California at Berkeley Berkeley California

**Keywords:** advanced biotechnology, bioassay development, digital cell biology, drug discovery, nanoscale cell culture, optical manipulation techniques, single cell technology

## Abstract

The new and rapid advancement in the complexity of biologics drug discovery has been driven by a deeper understanding of biological systems combined with innovative new therapeutic modalities, paving the way to breakthrough therapies for previously intractable diseases. These exciting times in biomedical innovation require the development of novel technologies to facilitate the sophisticated, multifaceted, high‐paced workflows necessary to support modern large molecule drug discovery. A high‐level aspiration is a true integration of “lab‐on‐a‐chip” methods that vastly miniaturize cellulmical experiments could transform the speed, cost, and success of multiple workstreams in biologics development. Several microscale bioprocess technologies have been established that incrementally address these needs, yet each is inflexibly designed for a very specific process thus limiting an integrated holistic application. A more fully integrated nanoscale approach that incorporates manipulation, culture, analytics, and traceable digital record keeping of thousands of single cells in a relevant nanoenvironment would be a transformative technology capable of keeping pace with today's rapid and complex drug discovery demands. The recent advent of optical manipulation of cells using light‐induced electrokinetics with micro‐ and nanoscale cell culture is poised to revolutionize both fundamental and applied biological research. In this review, we summarize the current state of the art for optical manipulation techniques and discuss emerging biological applications of this technology. In particular, we focus on promising prospects for drug discovery workflows, including antibody discovery, bioassay development, antibody engineering, and cell line development, which are enabled by the automation and industrialization of an integrated optoelectronic single‐cell manipulation and culture platform. Continued development of such platforms will be well positioned to overcome many of the challenges currently associated with fragmented, low‐throughput bioprocess workflows in biopharma and life science research.

## 1 INTRODUCTION OF LIGHT‐INDUCED ELECTROKINETICS AND IMPACT ON BIOLOGICS DISCOVERY

A new generation of techniques based on forces exerted by a light beam (known as optical manipulations) is enabling interactive biology at the cellular level, thus opening new opportunities in drug discovery. Optical manipulation—which enables highly selective and dynamic processes in micro‐ and nanoscopic systems—has proven to be a versatile and integrated technology throughout many scientific areas. This technology is based on light‐induced electrokinetics that gives rise to designated forces on both solid and fluidic structures. Since the discovery of the optical gradient and scattering forces in 1970 by Ashkin et al. (1970) a wide variety of optical manipulation methods have been developed including optical tweezers, plasmon‐based optical trapping/plasmonic tweezers, and optoelectronic tweezers (OET).

**Figure 1:**
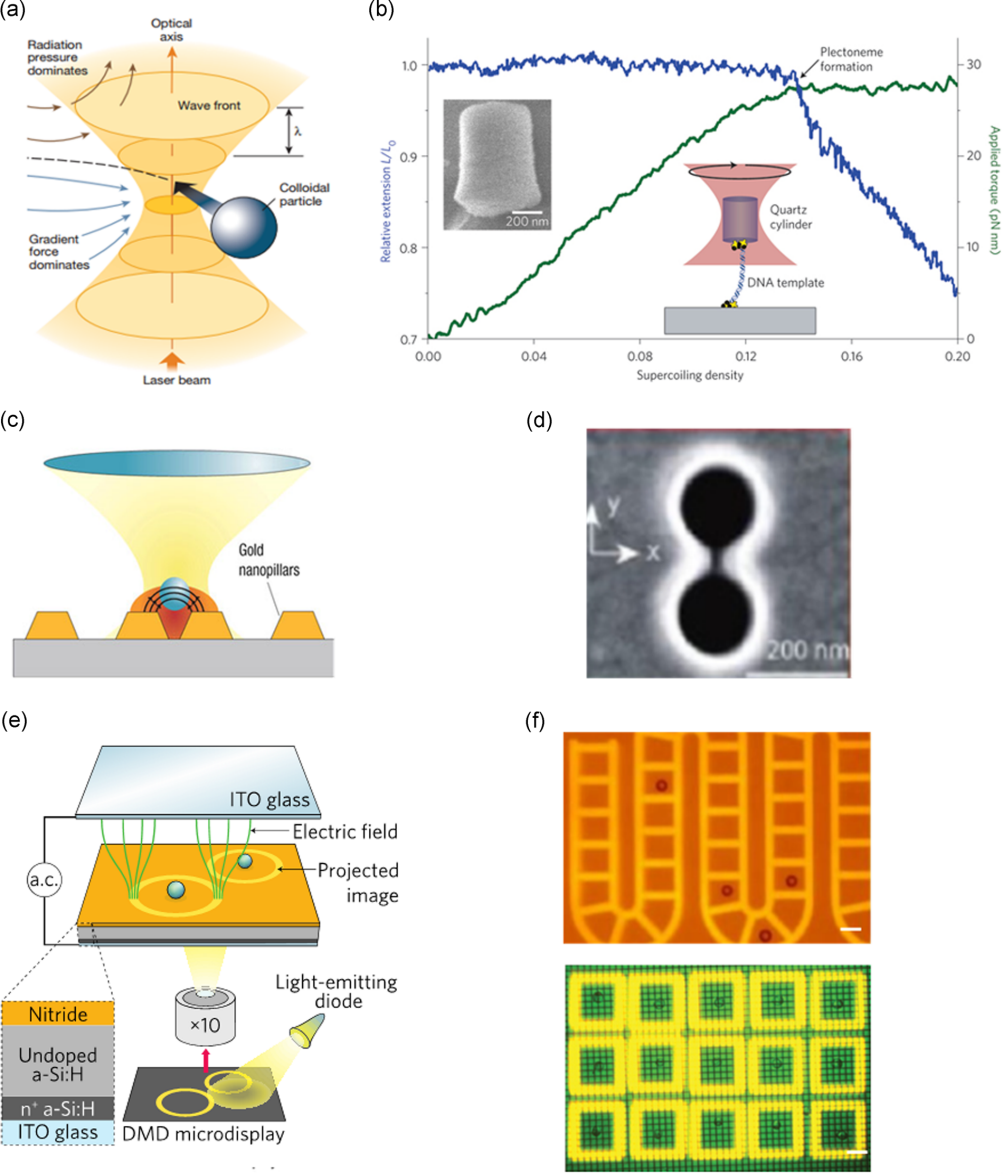
Optical manipulations | (a) Schematic of single‐beam optical tweezers: Particles as small as tens of nanometers are trapped by an optical gradient force generated via a high‐intensity laser (10^5^ to 10^7^ W/cm^2^; Grier, 2003). (b) Example of a nucleic acid system studied using optical tweezers, showing nanomechanical properties of filaments subjected to twist: The relative extension of DNA is monitored as trapped DNA is twisted with an optical torque wrench. The coiled DNA undergoes a phase transition from a twisted to a plectonemic form approximately 0.14 supercoiling density (Fazal & Block, 2011). (c) Plasmonic tweezers schematic: Patterned gold nanopillars give rise to localized surface plasmons causing a strong field enhancement under the trapping beam and suppression of the Brownian motion (characteristic of POT) resulting in improved particle confinement (Reece, 2008). (d) Molecular manipulation via plasmonic tweezers: A single bovine serum albumin (BSA) protein is trapped in the gap of a double hole nanostructure (Pang & Gordon, 2011). (e) Schematic of an optoelectronic tweezer device consisting of: a photoconductive layer of hydrogenated amorphous silicon (a‐Si:H) on an indium tin oxide (ITO) coated glass substrate (bottom layer), a liquid containing microparticles is sandwiched between the bottom layer and the top ITO‐coated glass layer. An AC electrical signal between the top and bottom layers in combination with patterned illumination create a nonuniform electric field that results in particle manipulation via dielectrophoresis (DEP; M. C. Wu, 2011). (f) Top image shows 20‐micron polystyrene particles confined in patterned light cages. Bar = 20  μm; Bottom image of shows virtual electrode cages (yellow) formed from projected light pattern to precisely control the position of individual cells. Bar = 50 μm (Hsu et al., 2010; M. C. Wu, 2011). POT, plasmonic optical tweezers

Optical tweezers utilize radiation pressure and gradient force from a single laser beam, focused by a high numerical aperture microscope objective, to trap and manipulate micro‐sized particles with forces at piconewton scales and nanometer range distances, (Figure 1a; Ashkin, 1970; 1992; Ashkin, Dziedzic, Bjorkholm, & Chu, 1986; Grier, 2003). Through the use of holographic optical tweezers, the ability to manipulate multiple particles in parallel has been enabled and advanced by the Grier and Dufresne labs (Curtis, Koss, & Grier, 2002; Dufresne, Spalding, Dearing, Sheets, & Grier, 2001; Mejean, Schaefer, Millman, Forscher, & Dufresne, 2009; Polin, Ladavac, Lee, Roichman, & Grier, 2005). As a result, optical tweezers have become a primary methodology for a variety of physical, chemical, and biological experiments. In particular, their capability to achieve highly accurate measurements on spatial (sub‐nanometer) and temporal (sub‐millisecond) regimes has ranked them as one of the forefront single‐molecule manipulation techniques with a wide range of applications (Fazal & Block, 2011; Moffitt, Chemla, Smith, & Bustamante, 2008; Neuman & Block, 2004; Neuman & Nagy, 2008; Pang & Gordon, 2011). Figure 1b shows an example of optical tweezers applied to the study of nanomechanical properties of double‐stranded DNA (Fazal & Block, 2011). A thorough review of the theory and practice of optical tweezers across multiple size scales and applications has been presented by Polimeno et al. (2018).

To further extend nanoscale optical trapping and overcome the diffraction limitations on spatial confinement associated with optical tweezers, plasmonic optical tweezers (POT) were developed (Juan, Righini, & Quidant, 2011; Miao & Lin, 2007; Reece, 2008; Shoji & Tsuboi, 2014). This approach combines optical tweezers with nanostructured gold substrates resulting in localized surface plasmons (Figure 1c). The tightly spaced surface plasmons generate a strong electric field enhancement and radiation pressure and enable stable optical trapping of particles at the nanoscale range. POTs facilitate efficient trapping of nanoparticles with laser intensities weaker than conventional optical tweezers (Figure 1d). In addition, the tailored design of the nanostructured substrates allows precise nanoscale control of the motion of nanoparticles.

Conventional and plasmonic optical tweezers have become powerful tools in biology allowing high‐resolution experiments on trapped single cells. However, both are limited to a small manipulation area due to large optical intensity requirements generated via high‐power lasers (at least 10^4^ W/cm^2^)^11^. In addition, the high optical power density causes cell damage over time and limits the duration of experiments. To overcome these limitations, a novel optical manipulation technique known as OETs was developed. OET enables massively parallelized optical manipulation of single cells via the utilization of optical beams to generate patterned virtual electrodes on a photoconductive material (Chiou, Ohta, & Wu, 2005; Hsu et al., 2010; M. C. Wu, 2011). OET operates within a much lower optical intensity (3 W/cm^2^) over a much larger addressable area (up to 11 mm^2^ as demonstrated on commercially‐available OET platforms (Berkeley Lights Inc.) with the installed Nikon 4×, 0.28NA objective.

A schematic of OETs is shown in Figure 1e demonstrating their operating principle (M. C. Wu, 2011). Structurally, it consists of a top transparent indium‐tin‐oxide (ITO) electrode and a bottom photoconductive hydrogenated amorphous silicon (a‐SI:H) electrode, separated by a fluidic chamber. The combination of patterned illumination and an applied AC bias between the two electrode layers generates a localized electric‐field gradient. Specifically, the electric‐field gradient results from the many orders of magnitude increase in conductivity of the a‐Si:H electrode layer when illuminated. This results in the creation of electron‐hole pairs in the electrode layer causing the voltage to drop across the fluidic chamber. In turn, objects such as particles or cells, experience dielectrophoresis (DEP) force in the presence of the light‐induced electric‐field gradient. The force $F_{\text{DEP}}$ applied to a particle with radius a is proportional to the electric field E applied (Zhang, Nikitina et al., 2018). The force can be positive (attractive) or negative (repulsive) depending on the relative values of the complex permittivity of the media $\epsilon_{m}^{⁎}$ and particle $\epsilon_{p}^{⁎}$ (Q. Chen & Yuan, 2019).

FDEP = 2πa3ϵmRe[ϵp⁎+ϵm⁎ϵp⁎−2ϵm⁎]∇(Erms)2

Thus, OETs enable full control of the position and motion of particles in the fluidic chamber by changing the light pattern through a digital light projector. An “optical conveyor belt” is shown in the top of Figure 1f. The OET with amorphous silicon photoconductor can only operate in media with conductivity <0.1 S/m. Typical cell culture media or physiological buffers have conductivities of approximately 1.4 S/m. To overcome this limit, the amorphous silicon photoconductor is replaced by single crystalline phototransistor (M. C. Wu, 2011). The phototransistor has 500x higher conductivity than amorphous silicon when illuminated. This allows long‐term culturing of cells and direct observation of the heterogeneity of doubling rates among the cells, bottom of Figure 1f (Hsu et al., 2010). As shown in Figure 1f, high‐resolution patterned illumination results in accurate single‐cell encapsulating compartments, which can also define their motion. OETs overcome many limitations associated with other light‐based techniques for micro‐particle manipulation, particularly in biological applications. First, OETs require a significantly low optical power (10^−1^ W/cm^2^) in comparison to conventional and plasmonic tweezers (10^4^–10^6^ W/cm^2^), making them noninvasive to achieve cell control and motion without compromising cell viability. Secondly, the low optical power requirements eliminate the necessity of a high numerical aperture objective to tightly focus a high‐power laser, which otherwise limits the area of particle control and manipulation. OETs significantly increase the manipulation area by two orders of magnitude in comparison to optical tweezers via the utilization of a 10x objective and a light‐emitting diode (LED) as the illumination source, thus facilitating high‐throughput processes (Chiou et al., 2005). Thirdly, the advancement from structural to high‐resolution virtual electrodes enables massively parallel and dynamic single‐cell manipulation.

## 2 BIOLOGICAL APPLICATIONS OF OETs

**Figure 2:**
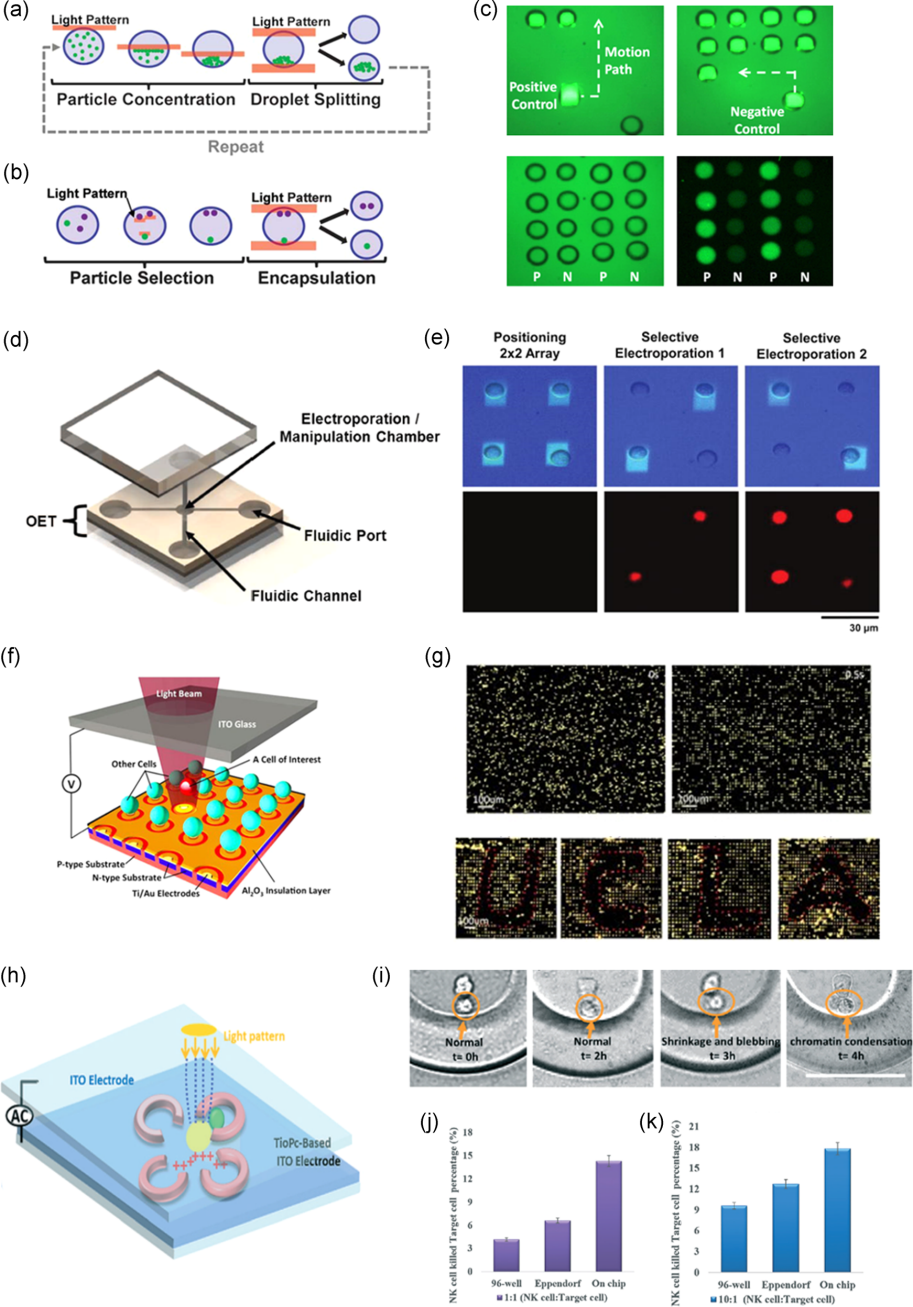
OET Bench‐scale applications | (a) Schematic of serial particle concentration: A light pattern is swept across the device concentrating beads at one end of the droplet via OET, followed by droplet splitting via OEW. Reiteration of this process results in an exponential increase in particle concentration (Valley et al., 2011). (b) Schematic of single cell selection and encapsulation: Patterned lights move specified cells in opposite sides of the droplet via OET, followed by droplet splitting via OEW resulting in a droplet with the single cell of interest (Valley et al., 2011). (c) A 4 × 4 array of droplets is formed by selectively positioning eight positives and eight negative controls. The negative control droplets contain blank viral transport medium while the positive control droplets contain 1.45 × 10^3^ viral‐particles/nl. No cross‐contamination was observed in the fluorescent image of the polymerase chain reaction (PCR) amplified products (Pei et al., 2015). (d) Schematic of light‐induced electroporation device: a top substrate is coated with the transparent conductor indium tin oxide (ITO), a middle layer consists of patterned SU‐8 layer defining the channel geometry, while bottom substrate coated with ITO and a photosensitive film (a‐Si:H; Valley et al., 2009). (e) First cells are positioned in a 2 × 2 array via low bias OET (0.2 kV/cm), first column, followed by a high bias (1.5 kV/cm) two‐step electroporation of specific cells. Fluorescent images show that only selected cells were electroporated, resulting in selective intracellular delivery of the membrane impermeant PI dye (Y. Yang et al., 2016). (f) Schematic of self‐locking optoelectronic tweezers (SLOT) consisting of a high‐density array of ring‐shaped electrodes, each controlled via an underlying phototransistor (Y. Yang et al., 2016). (g) Polystyrene beads (8‐μm) trapped in parallel on a 1 cm^2^ SLOT platform with more than 250,000 phototransistor traps. Initially, the particles are randomly distributed, next applied AC voltage between the top and bottom layer results in particle self‐locking into nearby traps. A scanning light beam selectively releases trapped particles, as shown through the formation of four letters – UCLA (Y. Yang et al., 2016). (h) Schematic of TiOPc‐based immunotherapy µ‐environment LabChip: consisting of PEG‐DA hydrogel four‐leaf‐clover‐shape (FLCS) microwells to generate a biomimetic environment (Ke et al., 2017). (i) Real‐time analysis of NK cell's behavior. Representative images showing NK cell and target cell morphologies over time to track immune cell cytotoxicity (Ke et al., 2017). (j) Percentage of target cells killed at a 1:1 ratio of NK cells: Target Cells (Ke et al., 2017). (k) Percentage of target cells killed at a 10:1 ratio of NK cells: Target Cells (Ke et al., 2017). NK, natural killer; OET, optoelectronic tweezer; OEW, optoelectrowetting; PI, propidium iodide

The utility of OET for particle and cell manipulations has led to a wide variety of advanced OET‐based devices and biological applications. Different approaches in the utilization of OET at the on‐the‐bench level resulted in a wide variety of applications including: manipulation of nano‐ and micro‐beads (Glaesener, Esseling, & Denz, 2012; Hsu et al., 2010; Ohta et al., 2007; Ota, Wang, Wang, Yin, & Zhang, 2013; Valley, Pei, Jamshidi, Hsu, & Wu, 2011; Williams, Kumar, Green, & Wereley, 2009), droplets (Pei, Valley, Wang, & Wu, 2015), nanowires (Jamshidi et al., 2008), and diverse biological cells (Jeorrett et al., 2014; Neale et al., 2009; Ohta et al., 2010; Park, Teitell, & Chiou, 2010; Shah, Ohta, Chiou, Wu, & Kim, 2009; Valley et al., 2009; Verma et al., 2014; Y. Yang, Mao, Shin, Chui, & Chiou, 2016). Figure 2 displays three examples of forward‐thinking OET‐based platforms.

Valley et al. (2011) developed a versatile device facilitating on‐chip particle and droplet manipulation via the integration of OETs with optoelectrowetting. This unified platform enabled serial particle concentration, resulting in an exponential increase in particle concentration that can be utilized for on‐chip sample concentration/purification (Figure 2a). In addition, the device has the ability to encapsulate single cells in microscale droplets of cell culture media, paving the way towards cell manipulation and analysis in a more relevant environment when compared with previous electrowetting concepts that required low conductivity buffer (Figure 2b; Hsu et al., 2010; Valley et al., 2011). A proof‐of‐concept experiment demonstrated the unified platform's ability to form a high‐density array of droplets and was applied in the biomedical application of viral detection (Figure 2c; Pei et al., 2015). Another OET‐based device integrated with microfluidic channels and chambers has been developed for high‐throughput and high‐selectivity electroporation of individual cells (Valley et al., 2009). A schematic of the device is shown in Figure 2d: lithographically defined channels were integrated with the OET device enabling light‐induced electroporation, maintenance of viable cell cultures, and perfusion of different soluble reagents. Electroporation was performed on HeLa cells using the membrane impermeant dye, propidium iodide (PI; Valley et al., 2009). Initially, low OET bias (0.2 kV/cm) was used to position individual cells in specified locations, followed by application of high electroporation bias (1.5 kV/cm) to selected cells resulting in the intracellular delivery of the PI dye (Figure 2e). In addition to electroporation, single cell lysis using OET has also been demonstrated (Kremer et al., 2014; Witte et al., 2014). Lastly, a device based on a novel concept, Self‐Locking Optoelectronic Tweezers (SLOT), has shown promising results in scaling up single‐cell manipulation across a significantly larger area (Y. Yang et al., 2016). The device schematic, Figure 2f, shows a prototype array of ring‐shaped phototransistors that control particle/cell trapping. A unique feature of the SLOT platform is the Al_2_O_3_ (an insulating dielectric layer) coating the surface to partially drop the voltage, enabling the single‐cell self‐locking function in high conductivity media. A device consisting of 250,000 phototransistor traps over a 1‐cm^2^ area has been shown to enable simultaneous trapping of over 100,000 polystyrene beads. Furthermore, the device enables the selective release of trapped particle via a scanning light beam as shown in Figure 2g, resulting in the formation of four letters standing for UCLA (Y. Yang et al., 2016). Another recent improvement called patterned optoelectronic tweezers enables more flexible particle manipulation by exposing the electrode layer in specific patterns that will hold particles after the light source is removed (Zhang, Shakiba et al., 2018). Additional methods to retain particles after light removal include the use of microwells to observe the interactions between cancer cells and immune cells (Ke et al., 2017). The schematic diagram of the four‐leaf‐clover‐shaped chip, Figure 2h, depicts the design of the immunotherapy µ‐environment LabChip. The stable and static µ‐environment enabled the accumulation of secretions from natural killer cells for real‐time analysis of their behavior on adjacent cancer cells (Figure 2i‐k).

The advanced OET‐based devices described above offer a wide variety of potential applications, including single‐cell studies involving multiplexed environmental stimuli, high‐throughput, and high‐resolution genetic transfection, study of cell‐to‐cell signaling, tissue engineering, in vitro fertilization, immunotherapy, and beyond. However, these platforms have been limited to proof‐of‐concept experiments at the bench level.

## 3 INDUSTRIALIZATION OF PARALLELIZED SINGLE‐CELL MANIPULATIONS

**Figure 3:**
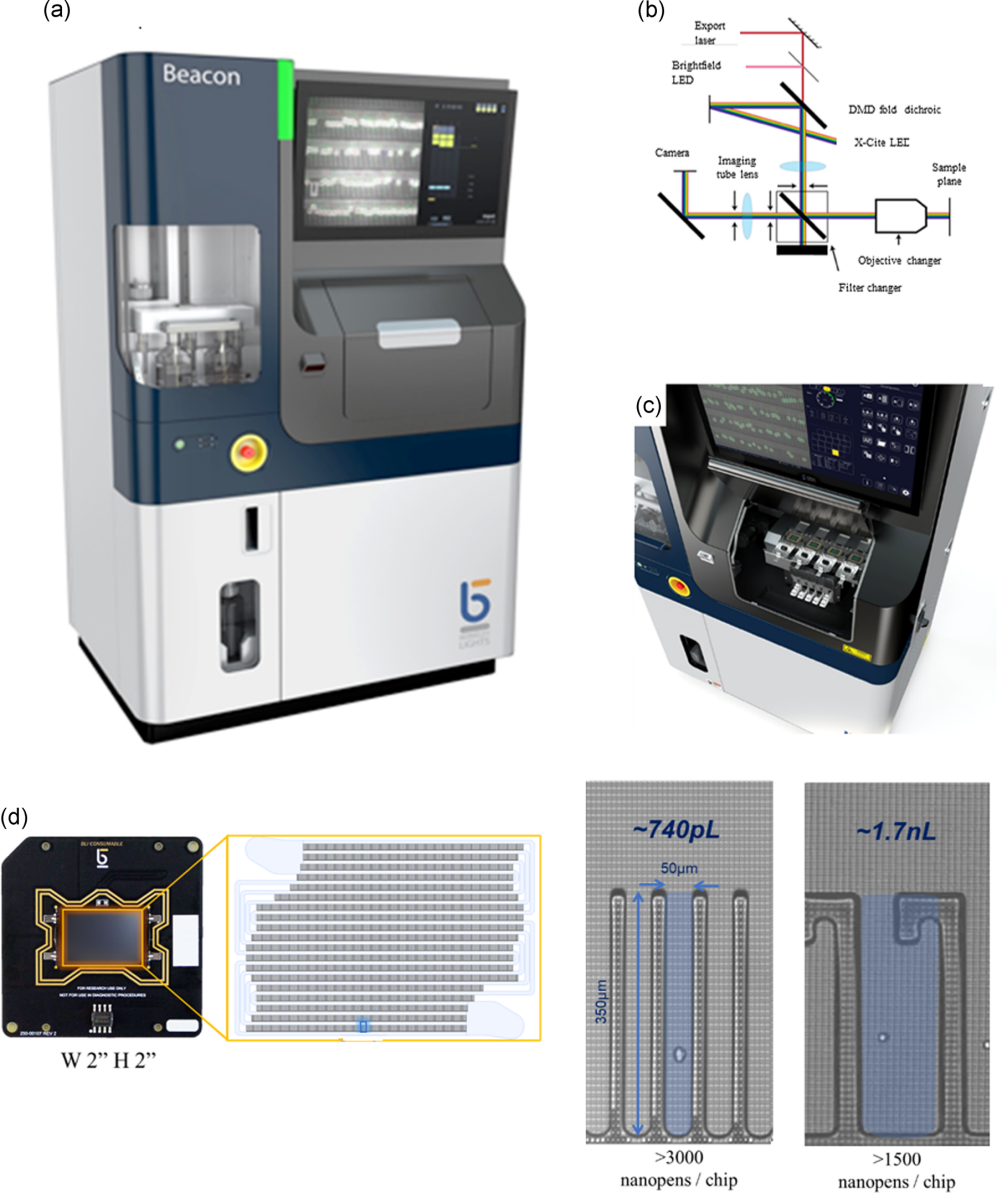
Platform technology: Beacon® | (a) Representative image of the instrument consisting of four bays: sample and chip, electronics, reagents, and waste. Dimensions: 42”W 32”D 72”H. (b) Schematics of the optical system: Camera: Andor Zyla; Filter changer: DAPI (Ex 390/40, DC BLTS‐0025), Em 452/45, FITC (Ex 475/50, DC 509‐FDi01, Em 540/50), TxRED (Ex 562/40, DC BLTS‐0026, Em 624/40), Cy5 (Ex 628/40, DC BLTS‐0027, EM 692/40), OEP (Ex 405R, DC 90R/10T, Em BLTS‐0030); Objective changer: Olympus Xfluor 4×/0.28 WD29mm, Nikon Plan Fluor 10×/0.30 WD 16 mm. (c) Sample bay hosting four nests, optics, camera, robotics, and plate incubators. (d) Representative image of a chip and the schematics of its fluidic structure. NanoPen structures associated with two different chip designs facilitate a variety of processes at different dimensions. OEP, opto‐electro‐positioning

The broad potential applications of OET have led to the development of an industrial platform, Beacon®, which incorporates opto‐electro‐positioning (OEP) technology, a variation on the OET technology, into an automated standalone system shown in Figure 3a. The system can be broken up into three key components: hardware, software, and consumables (chips and reagents). The hardware itself is approximately the size of a large refrigerator and requires power (120 V) and gas lines (CO_2_, house air, and optional O_2_) to operate, as well as an optional Ethernet port. An optical module consisting of an epifluorescent microscope with LED illumination and a custom digital mirror display for creating patterned illumination is situated above a motorized stage in the “sample bay” (Figure 3a,b). This three‐axis motorized stage holds four individual “nests” for housing up to four separate “chips.” Each nest has independently controlled temperature (15–40°C), using liquid‐cooled thermoelectric devices, as well as independent fluidic lines to control fluid exchange. Each nest has a dedicated syringe pump, located in a separate “reagent bay.” Each chip also has a “needle” that is independently actuated in the *z*‐direction for both input and output of media or reagents. A fifth syringe pump, also located in the reagent bay, is connected to a fifth input/output needle and can be used as a liquid handling robot. All of the input/output needles are situated above two “well plate incubators” (WPIs) in the sample bay. Each WPI acts as a miniature incubator, sized to hold a single 96‐well plate with temperature control (15–40°C) and is supplied with humidified air with specified CO_2_ and O_2_ content (0–20%). The WPIs hold separate 96‐well plates which provide both cells and reagents for importing into individual chips. These same WPIs are also used for exporting cells and are designed to keep cells in a suitable culture environment for at least 24 hr to maximize the flexibility of the system and eliminate the need for constant human supervision. The reagent bay holds the media used for on‐chip cell culture as well as reagents for cleaning in between runs. Below the reagent bay is the “waste module” for collecting all liquid waste.

Underneath the sample, bay resides the electrical components, which include the computer. Custom software controls all aspects of the system, with the main user interface being a large touchscreen monitor situated just above the sample bay. The chips are fabricated using semiconductor manufacturing technology whereby an array of phototransistors is patterned on a silicon substrate, which will become the floor of the microfluidic channels. Walls are created using photolithography and the ceiling consists of an ITO‐coated glass cover. These three layers are bonded together to form a chip which is mounted on a printed circuit board (PCB) that provides electrical connections as well as an EEPROM for storing identifying details for each chip.

Within each chip, there are a series of parallel fluidic channels with rows of NanoPen chambers situated along one side. The NanoPens are photolithographically defined structures that create unswept regions that are protected from shear flow, which can be created in the main fluidic channels by means of a corresponding syringe pump. These NanoPen chambers are connected to the main channels through a small opening (typically 50 µm wide and 40 µm tall). Due to the small scales, the inside of the NanoPens do not experience any convective mixing, rather any mass exchange between the channels and pens is dominated by diffusion. Figure 3d shows a chip with a representative fluidic structure, along with details on the two NanoPen structures that we have focused on for this study. The ability to create unswept chambers with nanoscale volumes that are still connected to a swept channel is crucial to creating a microenvironment suitable for robust cell growth while maintaining spatial separation of single cells or clones. Cells are much too large to effectively diffuse into or out of the NanoPens, but single cells can be deposited into individual chambers using light‐induced DEP. Once placed in a NanoPen, the cell remains unaffected by the shear forces in the main channel that are driven by the syringe pump; however, nutrients and/or waste products are free to enter/exit via diffusion, enabling a continuous perfusion culture system that enables simultaneous viewing of the growth of thousands of individual clones on a single chip.

## 4 APPLICATIONS IN BIOPHARMACEUTICAL INDUSTRY

The integrated technology has the flexibility and capability to enable a broad array of applications applicable to commercial large molecule drug development, including antibody discovery, clonal selection, gene editing, linking phenotype to genotype, and cell line development. We explored a wide variety of cell‐based discovery and optimization processes while simultaneously developing new assays focused on quantitative data readouts. In particular, we developed techniques for the manipulation and monitoring of isolated individual cells of yeast, hybridomas, B cells, Chinese hamster ovary (CHO), HEK293T, and HepG2. In addition, other groups have demonstrated the ability to select CRISPR edited clones as well as the ability to link phenotype to genotype, both on single cell levels and using T‐cells and tumor cells (Beaumont et al., 2018; Mocciaro et al., 2018). The process integration enabled by this platform technology has paved the way towards the acceleration of large molecule identification and optimization, relative to conventional methods and technologies.

## 5 ANTIBODY DISCOVERY

Traditionally, the discovery of mAbs derived from hyperimmunized animal models has relied on immortalization of the antibody‐secreting cell (ASC) via hybridoma technology (Akagi, Nakajima, Tanaka, & Kurihara, 2018; Basalp & Yucel, 2003; Y. Chen et al., 2018; Kohler & Milstein, 1975; Li et al., 2018), viral immortalization of the B cell directly (Kwakkenbos et al., 2010; Traggiai et al., 2004), or phage and yeast display of antibody fragments derived from cloning of the antigen‐experienced B cell mRNA repertoires (Chan, Lim, MacAry, & Hanson, 2014; Dong, Bo, Zhang, Feng, & Liu, 2018; Feldhaus & Siegel, 2004; Glumac et al., 2018; Grzeschik et al., 2019; Scholler, 2018). Although these methods are well validated for both drug discovery and reagent antibody generation, commercial‐scale application requires significant laboratory infrastructure, large‐scale plate‐based aseptic liquid handling automation platforms, and lengthy workflows dependent on mitotic doubling times to generate antibodies with commercial value. It is therefore obvious that antibody discovery techniques that enable direct B cell screening without the need for immortalization or transformation are of great interest. Direct B cell discovery techniques offer the potential to reduce development timelines, recover repertoires of functionally relevant IgG directly from B cells compartments not normally sampled (i.e., bone marrow), reduce or even eliminate altogether costly plate‐based aseptic liquid handling automation, and discover novel antibodies from species without traditional fusion partners (e.g. chickens, llamas, sharks). In addition, single B cell techniques that preserve the endogenous variable heavy (VH) and variable light (VL) pairing of the in‐vivo affinity matured IgG through the process, as opposed to high‐throughput DNA sequencing and rebuilding IgGs based on relative VH and VL frequencies (Reddy et al., 2010), may offer a more direct route to commercial antibody discovery.

A number of different single‐cell screening approaches have emerged over the years to address this opportunity. Comprehensive review papers describing these technologies have been published previously (Fitzgerald & Leonard, 2017; Seah, Hu, & Merten, 2018; Voigt, Semenova, Yamamoto, Etienne, & Nguyen, 2018), nonetheless brief introductions for a select few of these will again be presented here. FACS sorting of specific B cell subsets, plate‐based multiplex PCR, and recombinant antibody expression in suitable host cells have effectively recovered IgG sequences directly from select B cell subsets of both C57BL/6 wild‐type mice(Jin et al., 2009) and humans (Wrammert et al., 2008; X. Wu et al., 2010). Mettler‐Izquierdo et al. (2016) presented the gel encapsulated microenvironment (GEM) technique capable of interrogating up to 100 million B cells from hyperimmunized animals directly for antigen specificity. A relative of the fluorescent foci techniques (Babcook, Leslie, Olsen, Salmon, & Schrader, 1996; Tickle et al., 2009), the GEM technique encapsulates B cells of interesting in emulsion droplets using a mixture of agarose and dimethylpolysiloxane combined with either antigen‐coated beads and/or reporter cells expressing membrane antigens. Manual fluorescent microscopy is used to analyze the binding properties and localization of any secreted IgGs while physical recovery of the desired B cell is performed via manual picking using a micropipette. Microfluidic arrays generated via soft lithography microengraving have also been successfully used to isolate and screen monoclonal antibodies directly from B cell subsets without immortalization (DeKosky et al., 2013; Jin et al., 2009; Love, Ronan, Grotenbreg, van der Veen, & Ploegh, 2006; Story et al., 2008). These techniques are based on chip array technology combined with modifications to classical ELISPOT and/or ELISA techniques. In addition to isolating single cells at scales between 10^3^ to 10^5^, they offer the ability to screen directly against the antigen of interest and in some instances perform relative characterizations for key attributes such as binding affinity (Story et al., 2008). When combined with single cell PCR for VH and VL recovery and recombinant expression, panels of antigen‐specific monoclonal antibodies derived from these approaches can effectively be recovered within 1 to 2 weeks from the time of animal harvest. Nonetheless, not all labs are properly equipped to generate microarrays and for those that are, manual retrieval of the cells from these microarray systems still remains a fundamentally time‐consuming and highly specialized process.

Despite their innovative power and a diverse set of advantages over traditional hybridoma and display methodologies, there are a couple of common features that the above‐mentioned methods lack. The first is the ability to effectively culture nonimmortalized ASCs as single cells and maintain them in a viable antibody secreting state for multiple days. The terminally differentiated state of ASCs combined with their relatively fragile nature limits the amount of iterative IgG characterization that can be performed on a given set of ASCs. Recovering and recombinantly expressing IgGs that are lacking characterization data for key attributes such as binding affinity, cross‐species reactivity, or functional activity can translate into large and costly cloning and expression efforts and may ultimately fail at meeting the design goals for a specific discovery campaign. The second is the lack of an integrated system for digital record keeping of both images and raw numerical data from important processing steps in an antibody discovery workflow. Clonality and viability are key attributes in single cell workflows and of course, data integrity is a fundamental requirement for establishing intellectual property on novel inventions. A fully integrated direct B cell screening and recovery system that combines speed, sensitivity, characterization depth, ease of use, and workflow automation while simultaneously capturing digital records at each step of the screening workflow, therefore, could represent a quantum leap forward for commercial antibody discovery.

**Figure 4:**
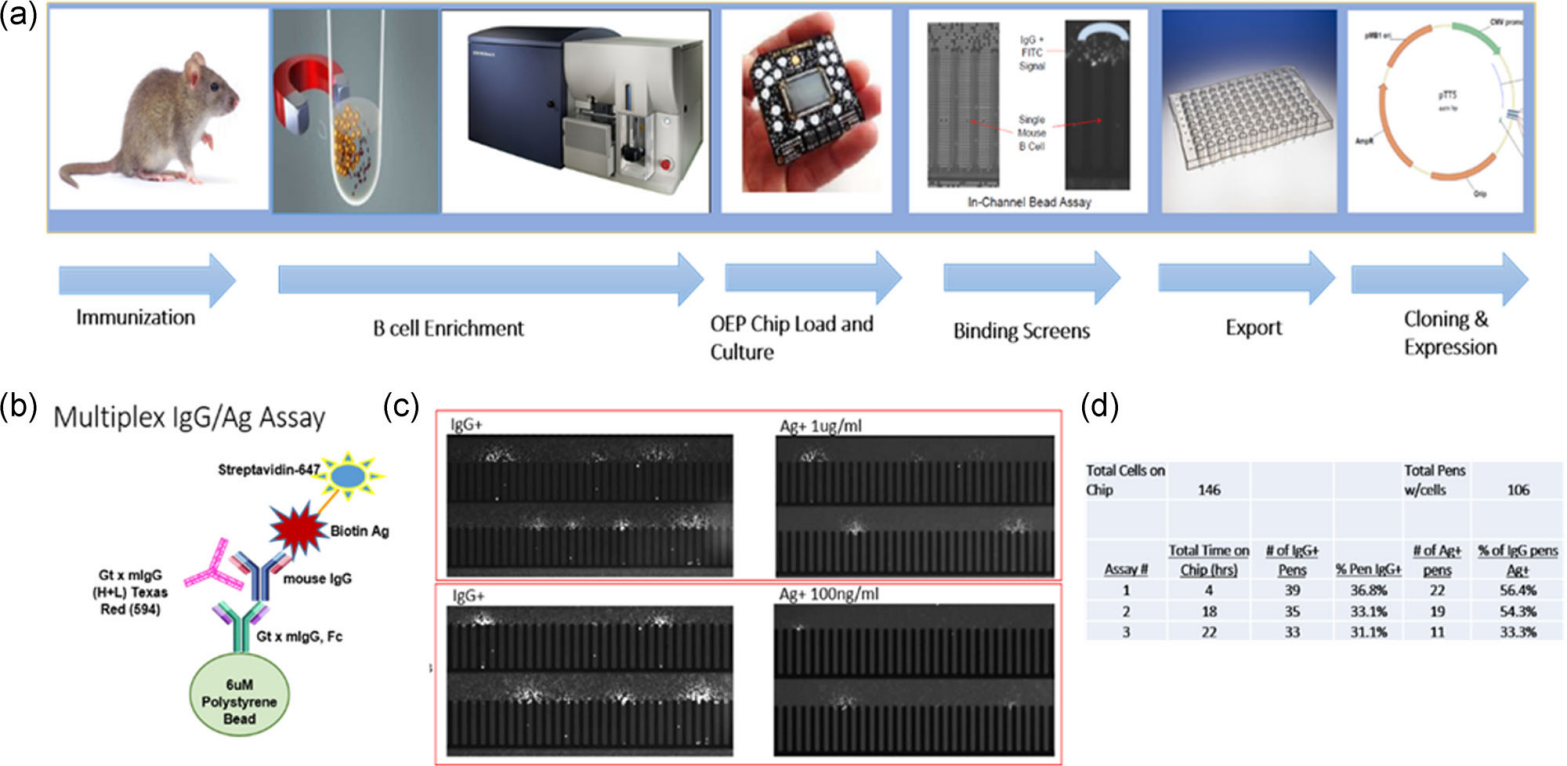
Primary ASC based antibody discovery | (a) Workflow overview: Immunization schedules designed to enhance the relative frequency of ASCs in specific compartments. Specific organ compartments were harvested, and ASCs enriched using magnetic bead negative selection followed by a multicolor FACS sorting strategy. Iterative multiplex screens were performed on the OEP platform. Selected hits were exported, the VH and VL sequences recovered via multiplex PCR, and the IgGs recombinantly expressed for further characterization. (b) Multiplex bead‐based assay design for soluble antigens: Bead size, antigen, secondary antibodies and detection fluorophore were chosen to specifically enable robust identification of antigen‐specific ASCs. (c) Images of IgG secreting (TRed) antigen‐specific (Cy5) B cells at two concentrations of antigen. (d) Percentages of IgG+ and antigen+ wells identified from enriched normal mouse ASCs as a function of time on chip. ASCs, antibody‐secreting cell; OEP, opto‐electro‐positioning; PCR, polymerase chain reaction

Using the Beacon platform technology, we have developed a nanofluidic optoelectronic  B lymphocyte screening technique (NanOBlast) that enables direct screening of secreted immunoglobulins from ASCs harvested from hyperimmunized wild‐type mice (Figure 4; Winters (2019). *Rapid Single B Cell Antibody Discovery Using Nanopens and Structured Light.* Manuscript submitted.). The process includes a magnetic negative selection to remove non‐B cells and a FACS‐based positive selection enrichment protocol to enrich for ASCs to address the low‐frequency distribution of antigen‐specific ASCs found in murine spleen and lymph node compartments. Using the tunable OEP parameters and customizable culture conditions on the platform, we effectively isolated single ASCs into nanopens while maintaining their viability. Within an hour of loading the ASCs, we executed two color soluble antigen bead‐based assays screening assays to simultaneously identify IgG producing and antigen‐specific ASCs. The 0.74 nanoliter volume of the nanopens in the OptoSelect™ 3500 chip combined with ASC secretion rates in the 10–1000 s of IgG/sec (Henn et al., 2009) allowed antigen‐specific IgG detection of most ASCs within 10 to 15 min. Exploiting the rapid turnaround time of the assays and the continued viability of the ASCs in the nanopens, we ran a second screen with a reduced concentration of the antigen to identify those ASCs secreting IgG with higher relative affinity. Selected ASCs were then exported out of the chip using the OEP and precision pump system of the platform directly into 96‐well plates containing lysis buffer. VH and VL sequences were recovered using a modified 5′ RACE and expression constructs synthetically built for downstream cloning, recombinant expression, and thorough antigen binding characterization.

Using the Beacon platform, single ASCs of interest can be effectively imported, cultured, screened and exported from the NanoPens of the microfluidic chip with precise control. Depending on the degree of characterization desired, ASCs can be cultured on‐chip for multiple days while iterative screening assays are executed. Although the data presented here only shows two back‐to‐back assays with two color multiplexing, one can imagine methods to expand the on‐chip characterization. For example, iterative four color multiplex bead assays could profile an individual ASC for reactivity to human, cyo, mouse and rat versions of a protein and so across multiple concentrations. Perhaps more exciting is using the microfluidic culture abilities of the Beacon to perform on‐chip assays directly for antibody function. Coculture experiments interrogating ligand blocking, signal transduction, receptor internalization, and agonism of functional target cells could potentially be of great value. As the Beacon technology matures, it will undoubtedly offer unique opportunities to transform antibody discovery workflows for both reagent as well as therapeutic applications.

## 6 OPTIMIZATION OF LARGE MOLECULES

The development of large molecule therapeutics with optimal potency, selectivity, and biophysical properties often requires the generation and screening of many engineered antibody variants (Chiu & Gilliland, 2016). Depending on the properties desired in the final molecule, these engineering designs may target affinity or selectivity modulation (Kiyoshi et al., 2014; Sellmann et al., 2016; Tiller et al., 2017), reduction in immunogenicity (Presta, 2006), better pharmacokinetics (Haraya, Tachibana, & Igawa, 2019; Hotzel et al., 2012), removal of chemically labile sites (Chelius, Rehder, & Bondarenko, 2005; DiCara et al., 2018; Haberger et al., 2014), and/or improvement in other biophysical properties related to manufacturing (Jarasch et al., 2015; Seeliger et al., 2015; Xu et al., 2013). Standard methods of screening panels of molecules that have been engineered for multi‐parameter optimization require cloning, expression, and purification of a sufficient quantity of protein to run multiple assays. This workflow is labor‐intensive and limits the number of designs that can be tested in a reasonable amount of time (Barnard, Hougland, & Rajendra, 2015; Bos et al., 2014; Estes et al., 2015; Schmitz et al., 2019; Winters, Chu, & Walker, 2015; X. Yang et al., 2013; Yoo, Provchy, Park, Schulz, & Walker, 2014).

**Figure 5:**
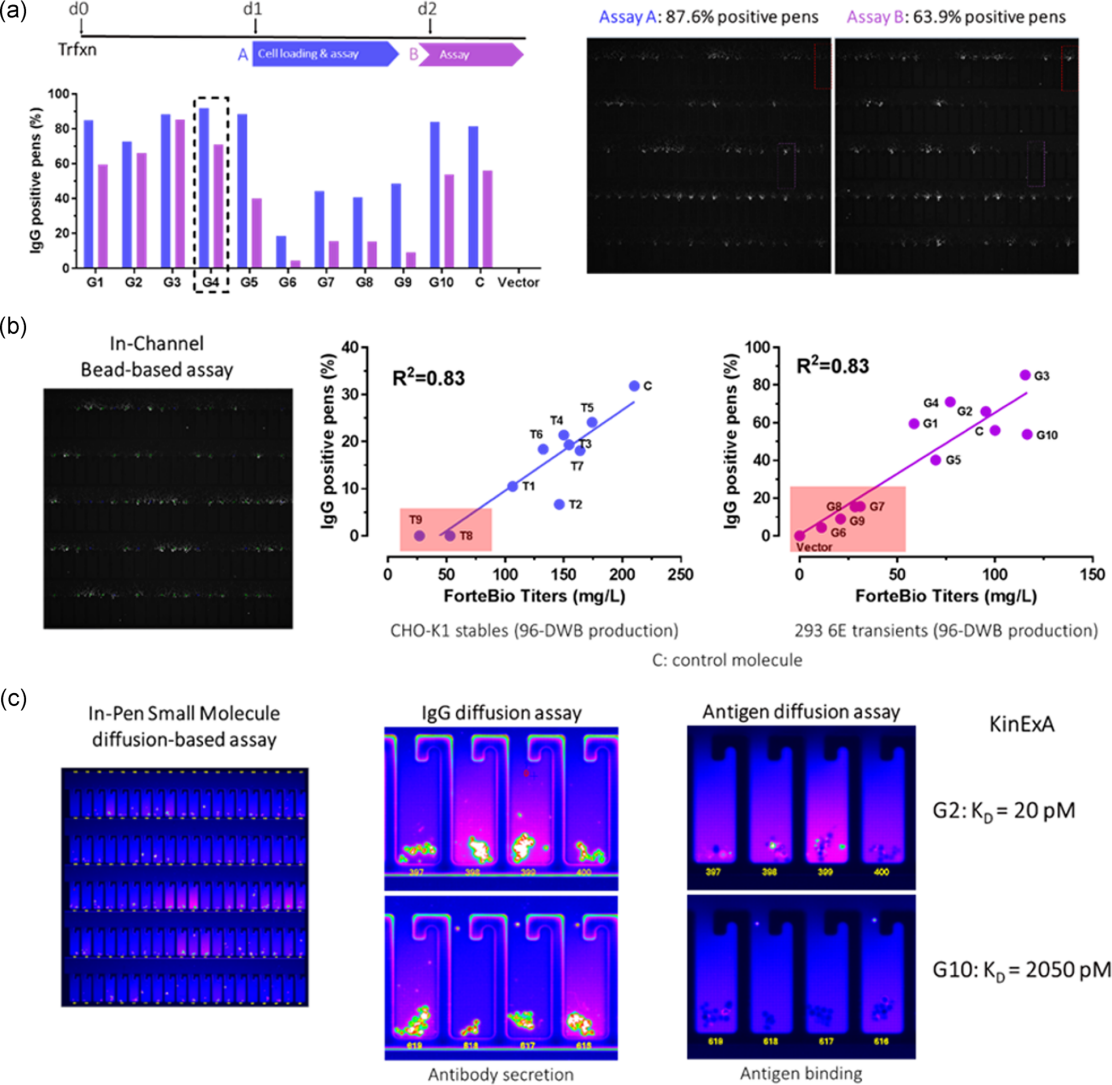
Engineered antibody screening | (a) Optimization of the transient process demonstrates robust detection of secreted antibody 24 hr posttransfection. Bead‐based IgG assay was sequentially run 2 hr (Assay A) and 24 hr (Assay B) after cell loading to assess the antibody secretion levels over time. (b) Bead‐based IgG secretion rankings of 10 different anti‐G‐protein‐coupled receptors (GPCR) antibodies accurately correlate with the FortéBio titers of standard production cultures for both stable CHO and HEK 293‐6E cell hosts. (c) IgG‐ and antigen‐specific diffusion assays enable differentiation of high affinity vs. low affinity anti‐GPCR antibodies. Binding affinity is calculated via the assessment of the remaining amount of labeled peptide and antigen, pseudocolored in magenta. CHO, Chinese hamster ovary

To address these limitations, we explored the development of a prescreen process on the OEP platform based on automated manipulation and analysis of nanoliter‐scale expression cultures of transfected antibodies. Very rapid screening of transfected cultures is possible because the platform allows for loading multiple cells per pen in addition to single‐cell loading. Initially, we focused on reducing timeline and resources necessary for screening engineered mAb panels by implementing assays to quickly identify and eliminate poor expressors and poor binders. Two relative titer ranking (RTR) assays were developed, either diffusion‐based (in‐pen) or bead‐based (in‐channel), to evaluate IgG secretion levels of both transiently transfected HEK 293‐6E cells (Durocher, Perret, & Kamen, 2002) and stably transfected and selected CHO pools. The in‐pen assays are based on a fluorescently‐labeled peptide that binds specifically to human IgG1 Fc, Spotlight™ huIg (Berkeley Lights, Emeryville, CA). The Spotlight reagent is imported onto chips loaded with antibody‐expressing cells and then flushed away after a short incubation. Because mAb‐bound peptide diffuses more slowly than free peptide, the amount of fluorescence remaining in each pen after an optimized flush time is proportional to the amount of IgG secreted by the transfected cells. We also developed an in‐channel assay for IgG secretion in which polystyrene beads coated with goat anti‐human IgG are imported onto the chip along with a fluorescently‐labeled secondary antibody and monitored over time. Unlike the diffusion‐based assay, in which fluorescent images are acquired at a single time point after flushing out the free label, the bead‐based assay can report on the kinetics of IgG secretion from each culture through the acquisition of images at multiple time points. In both assay formats, bright field images are used to eliminate pens without cells, allowing accurate ranking of molecule expression by calculating the fraction of pens secreting IgG for each antibody variant (Figure 5b).

For ease of assay optimization workflow, our initial experiments were conducted using stable CHO pools that had been selected for the integration of the transgenes using resistance markers. However, approximately 8 to 10 days required for cell recovery would add significantly to the overall timeline for an engineered antibody screening process. Therefore, we focused our further development on transient transfection systems, which shortened the process timeline from 15 days to 3 days. In our HEK 293‐6E transient transfection process, we loaded cells 24 hr after transfection and succeeded in detecting secreted antibodies within 2 hr after cell loading and through the following 24 hr (Figure 5a). The on‐chip RTR assay results for IgG secretion from these rapid transient expressions correlate with mAb titer from standard expression cultures as measured by the FortéBio Octet® system, similar to the RTR rankings from stable CHO process (Figure 5b). Because the exquisite sensitivity of the assay delivers robust results at early time points, we are able to implement a transient workflow that significantly reduces the timeline for evaluation of relative expression levels of engineered variants.

To further expand the characterization capabilities of the platform technology for engineered antibodies, we also established a diffusion assay for binding affinity to target antigen. In the example shown in Figure 5c, we developed and optimized an assay for on‐chip specific antigen binding to the soluble extracellular domain (ECD) of a G‐protein‐coupled receptors (GPCR). We transiently transfected HEK 293‐6E cells with 10 different anti‐GPCR antibodies with known binding affinity and evaluated their ability to bind fluorescently‐labeled soluble antigen 24 hr posttransfection. We imported and incubated 1 µg/ml fluorescently‐labeled antigen for 25 min, flushed for 8 min, and assessed the amount of label remaining in each pen via image acquisition. As shown in Figure 5c, the on‐chip antigen diffusion assay for this panel of engineered antibodies showed a promising correlation with binding affinity measured by KinExA. The combined analysis of IgG secretion and antigen diffusion assays enables the differentiation of high affinity versus low‐affinity binders and very rapid deselection of the worst variants in an antibody panel. More quantitative rankings using time‐based analysis of the intensity level per specified location are currently in development.

We demonstrated that the integrated technology of the platform enables expressing and assaying engineered antibodies on the nanofluidic chip in a streamlined process. Assays are performed in a nanoliter scale, which allows for sensitive detection of mAb secretion within hours of cell loading. We showed IgG secretion rankings correlated with mAb titer from standard expression cultures, and we saw a promising correlation of on‐chip antigen binding with binding affinity from purified antibodies. Combined with a transient transfection process, our on‐chip assays shorten the timeline for screening IgG expression and binding to 3 days. These results suggest that the platform technology could potentially be used as a prescreen to quickly narrow down engineered panels by deselecting poor expressors and poor binders. To further develop mAb optimization on the platform, we are developing novel processes for *K*_d_ binding affinity assays, *k*_off_ kinetic assays, and cell‐based functional assays.

## 7 DEVELOPMENT OF A QUANTITATIVE SINGLE‐CELL ASSAY

**Figure 6:**
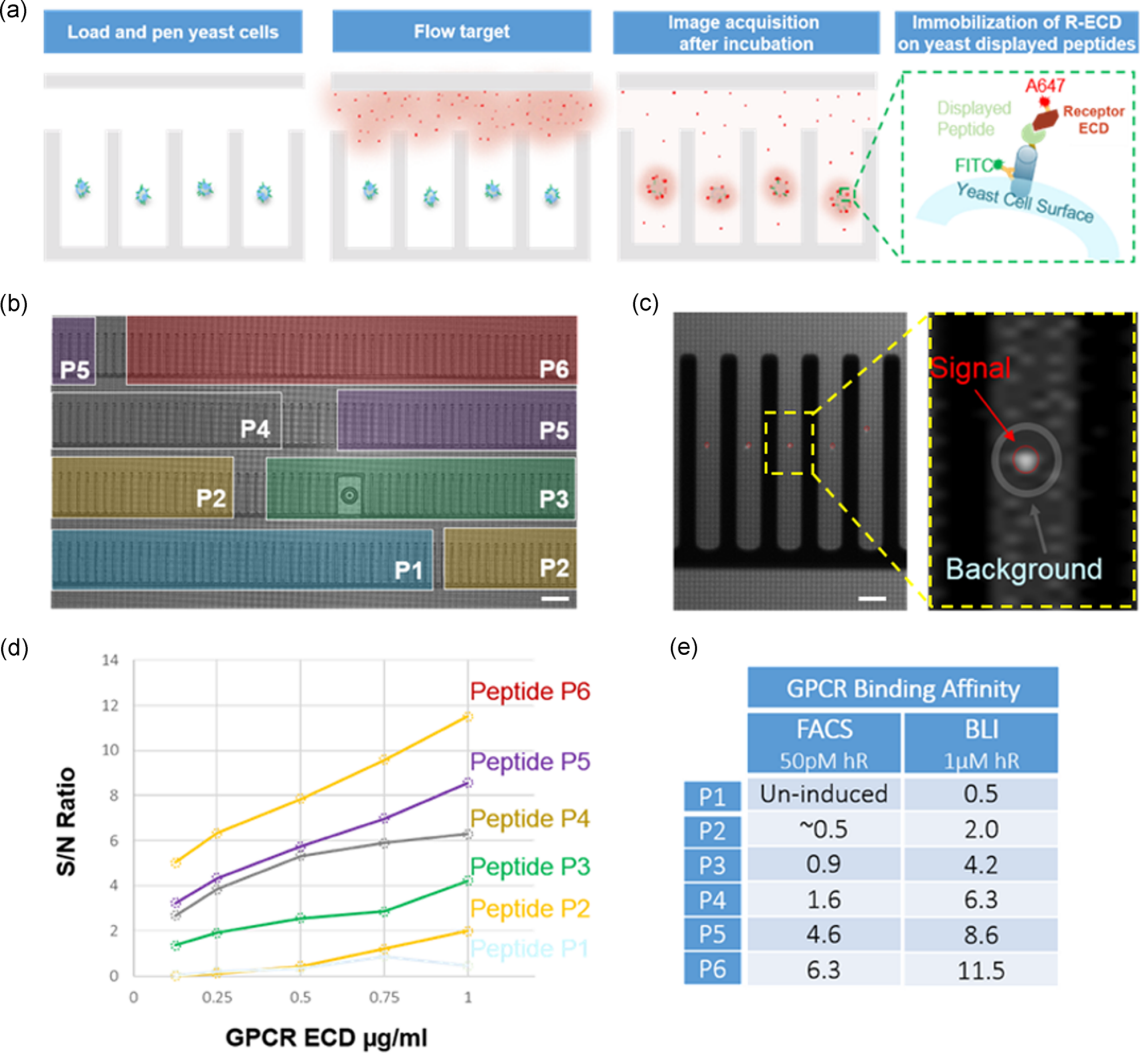
Single‐cell quantitative assay | (a) Workflow schematic: After loading, cells are identified and trapped into light cages facilitating single‐cell penning via OEP. Next, low concentration (0.125 µg/ml) of dye‐labeled (A647) target (receptor ECD) is imported and incubated for 10 min followed by multiplexed image acquisition (bright field, FITC, and Cy5). The process is reiterated at increasing target concentrations up to 1 µg/ml. (b) Loaded and penned six different yeast‐displayed peptides (P1–P6), previously induced, in specified chip region shown in different colors. Bar = 150 µm. (c) Representative fluorescent image used to quantify the concentration of bound receptor to peptides displayed on individual yeast cells; Schematic of the automated data analysis SNR: Fluorescence intensity of individual cells (circled in red) is normalized by the fluorescence intensity of the background via the creation of a doughnut region (gray) around each region of interest. Bar = 50 µm. (d) Relative ranking of binding affinity: S/N Ratio plotted versus a series of receptor ECD concentrations from 0.125 to 1 µg/ml. (e) GPCR binding affinity rankings (SNR) generated on the OEP platform ‐ based on individual cell analysis ‐ were consistent with conventional measurements (FACS). ECD, extracellular domain; GPCR, G‐protein‐coupled receptors; OEP, opto‐electro‐positioning; SNR, signal‐to‐noise ratio

To further expand the utility of the Beacon platform, we explored a variety of library formats, with yeast display as a primary focus due to its wide versatility to perform protein and peptide engineering (Cherf & Cochran, 2015). Yeast display has been used to discover de novo binders (McMahon et al., 2018; Wang et al., 2016), affinity mature existing binders (Tiller et al., 2017), engineer in pH‐sensitivity (Schroter et al., 2015), improve protein thermal stability (Jones, Tsai, & Cochran, 2011), and enhance enzyme kinetics (I. Chen, Dorr, & Liu, 2011). Our work used yeast‐displayed peptides isolated from an affinity maturation campaign against a receptor ECD to develop a binding affinity assay on the platform. The precise cell manipulation on the Beacon via OEP facilitated the development of a single‐cell assay even for small‐sized cells such as yeast (diameter 5 microns). The binding affinity assay is schematically summarized in Figure 6a. First, six different fluorescein‐labeled yeast displaying peptides were loaded and penned sequentially in specified chip regions, Figure 6b. Single cells were loaded in individual pens to enable accurate signal detection. Next, the lowest concentration of Alexa Fluor 647‐labeled receptor ECD was imported and incubated for 10 min. After incubation, the binding of receptor ECD to yeast‐displayed peptide was assayed through multiplex image acquisition: bright field ‐ displaying the cell position, FITC ‐ quantifying the concentration of displayed peptide, and Cy5 ‐ quantifying the concentration of the bound receptor. To measure the binding affinity, we ran a series of specified antigen concentrations sequentially from 0.125 to 1 µg/ml on the chip.

We developed internal analysis software to quantify the peptide affinity at the single‐cell level via signal‐to‐noise ratio (SNR) analysis, Figure 6c. Accurate quantification of relative binding affinity via SNR was based on integrated multiplexed signal readout and background analysis taking into account the fluorescent background from the unbound receptor ECD. Figure 6d summarizes the results of one of our initial experiments where we evaluated the binding of six yeast‐displayed peptides across a series of ECD concentrations. The relative affinity rankings measured on the OEP platform—based on individual cell analysis—were consistent with conventional FACS analysis of the same constructs. In addition, we achieved on‐chip induction of yeast library paving the way towards the development of on‐chip functional assays.

## 8 DEVELOPMENT OF GENOMIC APPLICATIONS

With the capabilities now available for single‐cell sequencing, integrated single‐cell analysis systems like this one will find many applications that link up genotype to phenotype. Two recent examples capitalize on this integration to generate and identify CRISPR edited clones very quickly in an automated manner as well as isolate rare cells for subsequent genotyping.

Mocciaro et al. (2018) demonstrated a method to identify, select, and expand cell lines with desired edits in less than 10 days, a process that would normally take 3 to 4 weeks using traditional subcloning techniques. Using the Beacon platform, they were able to load > 3000 single primary human T cells on an OptoSelect chip, after electroporation of the cell population with Cas9 RNPs. After 3 days of clonal expansion, clones were screened for the desired edits using a surface marker. The desired edit, in this case, was designed to replace 12 nucleotides in the *CXCR4* gene, which encodes for a transmembrane receptor protein clinically‐relevant for HIV infection. Homozygously edited cell lines should not express the marker and therefore a fluorescent assay using anti‐CXCR4 antibody was used to identify nonfluorescent clones for selection. Selected clones were exported with a process that splits each clone into two separate exports, one for sequencing and one for proliferation. Expected edits were confirmed by genomic DNA sequencing in some of the selected clones, demonstrating the ability to quickly identify edited clones of interest while simultaneously keeping a live culture ready for scaleup (Mocciaro et al., 2018).

Beaumont et al. (2018) used the nanofluidic technology in a similar way to connect phenotype to the genotype of single cells taken from ovarian cancer patients. After placing ovarian tumor cell lines in the chip, colonies of cells from each cell line were monitored for multiple phenotypes, such as growth rate and cell surface protein expression. Protein expression was measured by diffusing fluorescent antibodies into and out of the NanoPens, allowing for on‐chip fluorescent cell staining and phenotyping. Individual cells were also cloned and exported to well plates for subsequent processing and sequencing with a targeted hotspot sequencing panel thus demonstrating the compatibility of the platform with downstream nucleic acid sequencing workflows (Beaumont et al., 2018).

## 9 DEVELOPMENT OF CELL LINES FOR PRODUCTION

The generation of a highly productive manufacturing cell line is a key and important step in the development process for large molecule therapeutics (Estes & Melville, 2014; Fischer, Handrick, & Otte, 2015). To select a cell line that will give high productivity, long‐term stability, and consistent product and process consistency, subcloning and extensive screening is required on hundreds of clonal cell lines, measuring growth, productivity and product quality in multiple screening assays (Wurm, 2004). In addition, regulatory agencies require that clinical trials be initiated with material from clonally derived cell lines and banks, so documentation that the cell line is derived from a single cell is required to provide that assurance of clonality (Tharmalingam et al., 2018).

Cloning was historically accomplished by limiting dilution of stably transfected pools into 96‐well plates (Kim, Kim, & Lee, 2012). This method has a high rate of error and requires multiple rounds of cloning to satisfy clonality assurance required by regulatory agencies. This traditional limiting dilution approach provides no information on the productivity of each individual clones. Modern approaches use FACs to deposit single cells into 96 or 384 well plates followed by imaging of each well on the same day (DeMaria et al., 2007; Fieder, Schulz, Gorr, Bradl, & Wenger, 2017). FACs deposition into well plates provides both an increased assurance that a single cell is deposited into each well and can be coupled with surface staining for the protein of interest to enable isolation of only clones producing high amounts of the desired protein. However, several challenges remain. FACs exposes cells to high levels of pressure leading to reduced viability and recovery of isolated cells. Furthermore, surface staining methods identify protein levels retained in the plasma membrane, but the correlation between protein retained in the plasma membrane of the cell and amount secreted is not well established. This approach also only measures proteins levels at a single point in time, but protein synthesis, modification, and secretion are a highly dynamic process that is well known to change during the cell cycle (Alber & Suter, 2019). As a consequence of low recovery and poor‐quality screening, a typical cloning process will use 50 to 100 or more plates to isolate and screen hundreds to thousands of clones of single cell origin to find a suitable manufacturing line. Manually verifying clonality, transferring and scaling up clones from 96‐well plates production scale, and performing a bench‐scale production assessment experiment is an extremely resource‐intensive endeavor. Moreover, some conventional instruments that enable higher throughput screening of clones for cell line development are limited to a solid media format that may not be relevant to downstream culture conditions, while miniature bioreactors can provide relevant culture conditions but are limited to tens of clones at a time.

**Figure 7:**
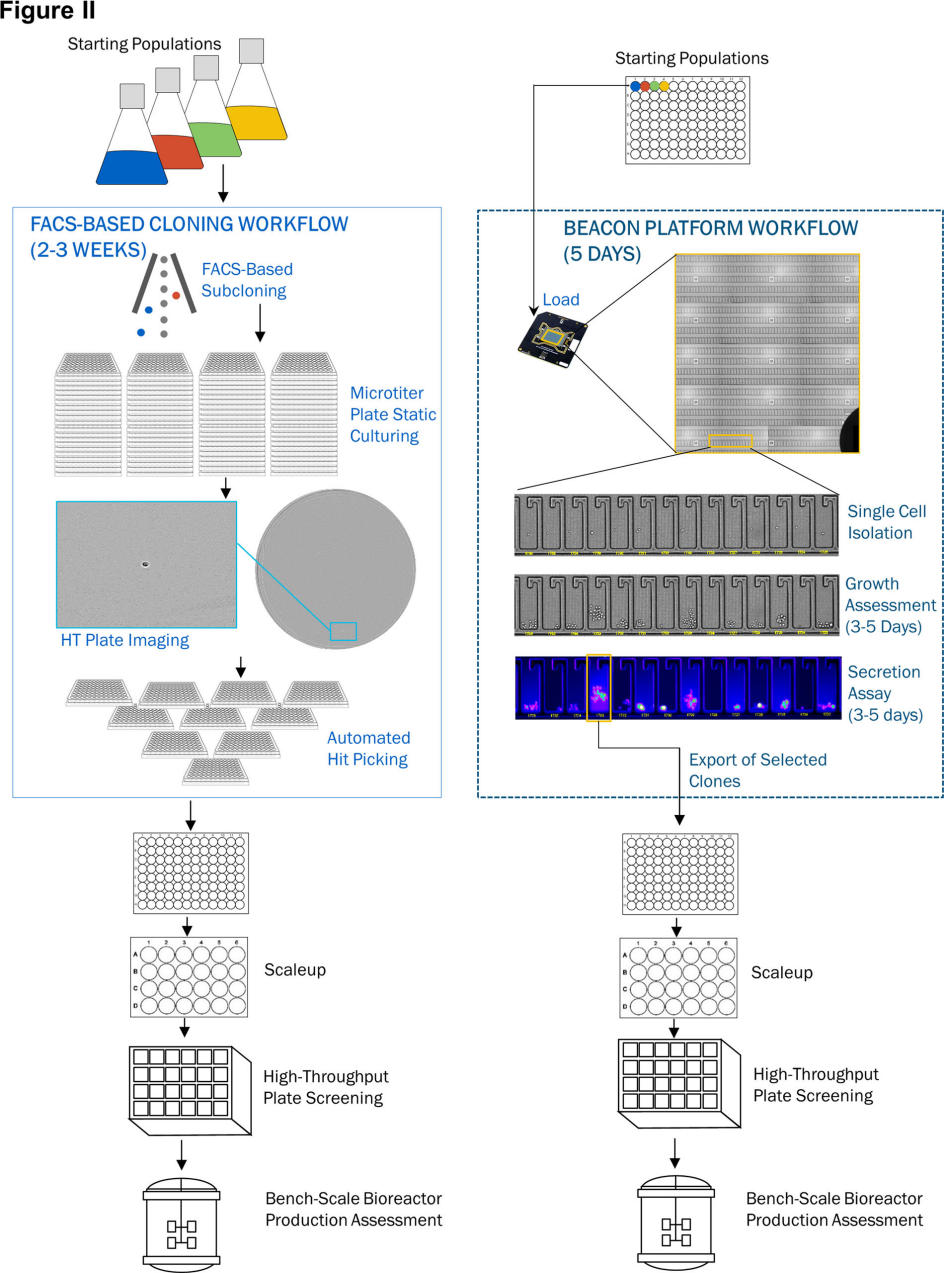
Comparison of a microtiter plate based cloning workflow vs. a nanofluidic chip subcloning workflow. A depiction of the steps involved in performing a clonal isolation and expansion workflow using two approaches. Differences are highlighted in boxes for FACS‐based workflow(solid) and platform workflow (dotted). Standard subcloning operation: A heterogeneous population is isolated and deposited into microtiter plates using FACS‐based cell sorting, followed with high‐quality, high‐throughput whole well imaging to verify a single cell in a well. After growth and repeated imaging, colonies are picked and consolidated using automation liquid handlers. Top clones are then screened in a bioreactor to select the final clone. In contrast, the platform workflow enables single cell isolation, growth assessment, and high‐throughput screen on the chip (dotted box) in the nanofluidic workflow, and only those clones that meet the desired criteria are exported and expanded for further evaluation (Le et al., 2018)

Nanofluidic technologies offer a promising solution to further miniaturize and increase the efficiency of manufacturing cell line generation (Sackmann, Fulton, & Beebe, 2014). Indeed, we have demonstrated that CHO cell lines can be isolated using the Berkeley Lights technology with high assurance of single cell origin, cultured, screened, and exported at high efficiency (Le et al., 2018). In a head to head comparison with a FACS‐enabled microtiter plate‐based workflow, we were able to generation of comparable clonal cell lines with reduced resources, summarized in Figure 7. Over all recoveries of the clonal CHO cell lines were shown to be higher than other methods, in part due to the improved conditions nanoscale culture offers. A rich data set is generated to support clonality, tracking, and population understandings to enable early decisions and identification of highly performing cell lines. Single‐cell data obtained from cell lines provide insights into defining population characteristics of the production cell lines that are not currently possible with the traditional FACS‐based methods, offering the potential to make improved cell line choices and better predict downstream success (Le et al., 2018).

## 10 CONCLUSION

Innovation in basic science has delivered some outstanding advancements to biomedical research over the last 40 years including transgenic mice, PCR, genetic sequencing, gene editing, and many others. Interestingly, most of these innovations were realized using relatively simplistic cell and liquid handling technologies invented in the 1950s: pipettes and well plates. In many cases, the pace of applied science and technology development has been outpaced by basic scientific progress. The traditional approach taken by those in the field has been to utilize straight‐forward technologies at the microscale level. These microscale approaches reach practical limits very quickly, as the complexity of rooms filled with single‐function equipment and automated robots still struggle with recent advances in research methodology, particularly on individual cells at the nanoscale fluidic regime. The promise of “lab on a chip” technology has been slow to mature towards industrial applications, but the promise remains the same: miniaturization of basic cellular manipulations should lead to faster and more efficient discovery, requiring less reagent and effort due to enhanced sensitivities. The platform technology discussed here, Beacon, overcomes such limitations through the capability to maintain physiologically‐relevant culture environments of thousands of segregated cells while performing numerous types of very sensitive assays all under reproducible computer control, otherwise known as “digital cell biology”. Importantly, this ability to flexibly string together multiple processes and tests (manipulate, grow, assay, interrogate, select) on a single system allows for full biological workflows to be performed with significantly minimized resources.

Digital cell biology on the platform creates the opportunity to transition many of today's cell‐based well plate assays to a unique new format, unlocking a step function increase in workflow scale and speed. Complex and time‐consuming assays, like the measurement of growth and IgG secretion rate on thousands of clones, have proven to be straight‐forward to transfer onto the platform's nanofluidic environment, making them routine to run on a day to day basis. We envision many more complex assays being reduced to routine software‐controlled workflows, such as on‐chip functional assays that require multiple precisely defined populations of cells interacting with reporter cell‐based assays. Examples include plasma B cells combined with reporter cells that indicate if the IgG molecule is functional, T cell activation by dendritic cells, T cell killing of tumor cells, or defined cell number micro‐organoids. In addition, we believe that capture of real‐time assay kinetics of single cells or cell clones across time and across multiple assay types for the same cell population will enable opportunities to discover and rank individual clones that previously relied on single snapshots of the population in time, like FACS and ELISA. Measuring cell surface protein expression, protein secretion, metabolic profile in addition to RNA‐seq from the same individual cell will enable researchers to build deep profiles or cell “fingerprints”, unlocking novel insights at the individual cell level. Combining the power of machine learning and AI with digital cell biology offers additional power to unlock deep insights into mechanistic biology that has thus far proven elusive at the macro scale. This technology can be applied to many areas within biopharma including antibody discovery, assay development, antibody engineering, and cell line development. With the ability to select the right cells, the technology can also be applied in the future to cell therapy manufacturing as well as workflows within synthetic biology, and diagnostics. We believe digital cell biology platforms will prove to be transformative to multiple industries that require a dramatic step forward in speed and scale to realize the next wave of breakthroughs.
